# Covid-19 in Brazil in 2020: impact on deaths from cancer and cardiovascular diseases

**DOI:** 10.11606/s1518-8787.2022056004040

**Published:** 2022-04-11

**Authors:** Beatriz Cordeiro Jardim, Arn Migowski, Flávia de Miranda Corrêa, Gulnar Azevedo e Silva

**Affiliations:** I Instituto Nacional de Câncer Coordenação de Prevenção e Vigilância Divisão de Detecção Precoce e Apoio à Organização de Rede Rio de Janeiro RJ Brasil Instituto Nacional de Câncer. Coordenação de Prevenção e Vigilância. Divisão de Detecção Precoce e Apoio à Organização de Rede. Rio de Janeiro, RJ, Brasil; II Universidade do Estado do Rio de Janeiro Instituto de Medicina Social Hésio Cordeiro Rio de Janeiro RJ Brasil Universidade do Estado do Rio de Janeiro. Instituto de Medicina Social Hésio Cordeiro. Rio de Janeiro, RJ, Brasil; III Instituto Nacional de Cardiologia Coordenação de Ensino e Pesquisa Núcleo de Epidemiologia Rio de Janeiro RJ Brasil Instituto Nacional de Cardiologia. Coordenação de Ensino e Pesquisa. Núcleo de Epidemiologia. Rio de Janeiro, RJ, Brasil

**Keywords:** COVID-19, Cardiovascular Diseases, Neoplasms, Comorbidity, Mortality

## Abstract

**OBJECTIVE:**

To analyze the impact of the covid-19 pandemic on mortality from cancer and cardiovascular diseases (CVD) as underlying cause and comorbidity in Brazil and Brazilian regions in 2020.

**METHODS:**

We used the 2019 and 2020 databases of the Mortality Information System (SIM) to analyze deaths occurring between March and December of each year that had cancer or CVD as the underlying cause or comorbidity. Deaths from covid-19 in 2020 were also analyzed. To estimate the Standardized Mortality Ratio (SMR) and the excess of deaths, 2019 data were considered as standard.

**RESULTS:**

Between March and December 2020, there were 181,377 deaths from cancer and 291,375 deaths from cardiovascular diseases in Brazil, indicating reduction rates of 9.7% and 8.8%, respectively, compared to the same period of the previous year. The pattern was maintained in the five Brazilian regions, with lower variation for cancer (-8.4% in the South to -10.9% in the Midwest). For CVD, the variation was greater, from -2.2% in the North to -10.5 in the Southeast and South. In the same period of 2020, these diseases were classified as comorbidities in 18,133 deaths from cancer and 188,204 deaths from cardiovascular diseases, indicating a proportional excess compared to data from 2019, of 82.1% and 77.9%, respectively. This excess was most significant in the Northern Region, with a ratio of 2.5 between observed and expected deaths for the two conditions studied.

**CONCLUSIONS:**

Excess deaths from cancer and CVD as comorbidities in 2020 may indicate that covid-19 had an important impact among patients with these conditions.

## INTRODUCTION

The covid-19 pandemic, a disease caused by the new coronavirus (SARS-CoV-2), has substantially changed the mortality profile in many countries. In 2020, approximately one million additional deaths occurred in 29 high-income countries compared to the previous four years^1^. In Brazil, several authors showed that, compared to previous years, in 2020 there was clearly an excess of deaths^2^ as well as an increase in the hospital mortality rate^6^.

Historically, from the second half of the last century, the burden of chronic non-communicable diseases (NCDs) took center stage, accounting for more than 60% of deaths worldwide today^7^. Cardiovascular diseases (CVD) and cancer have been the two main causes of death in Brazil for many decades.

Experimental and observational evidence shows a consistently higher risk of evolution to severe case of covid-19 in older individuals and individuals with comorbidities^8,9^ due to acute respiratory syndrome (SARS). Among patients with NCDs, such as cancer and CVD, health conditions may worsen as a direct or indirect result of the pandemic, either due to greater fragility or a worse response to infection by coronavirus type 2, which causes SARS (SARS-Cov-2), or to difficulty in accessing and continuing care of these chronic conditions in health services or due to impairment to actions of early detection, diagnostic confirmation and early treatment^10^. Consequently, assessing morbidity and mortality in individuals with increased risk of developing severe covid-19 is essential for determining preventive and therapeutic strategies for this specific group and for investigating the impact of the pandemic on their underlying diseases^11^.

The analysis of accessible information in the Brazilian Unified Health System (SUS) is essential for assessing how covid-19 has changed the country’s mortality profile, as well as for identifying situations aimed at improving health surveillance. The objective of this study was to analyze the impact of the covid-19 pandemic on mortality from cancer and CVDs, both as a underlying cause and as a comorbidity in Brazil and Brazilian regions in 2020.

## METHODS

The data on deaths occurred in 2019 and 2020 (preliminary basis available) used in this study were obtained from the Mortality Information System (SIM), on the homepage of the SUS Department of Informatics (Datasus)^a^. The general and regional population data were obtained from the population projections of the Brazilian institute of geography and statistics (IBGE), 2018 edition^b^.

The SIM has been improved since its creation in 1975, and its quality and coverage are considered good by international standards^12^. The data in the medical certification of cause of death (MCCOD) that feed the SIM are filled in by doctors and divided according to rules that allow determining the underlying causes of death, the antecedent and contributory causes (also known as comorbidities). The MCCOD form adopted in Brazil classifies the causes of death according to the World Health Organization (WHO) standard. Following the causal chain of events, the underlying cause is the disease or injury (or circumstances in the case of external causes) that initiated the chain of pathological events expressed in the antecedent causes and which directly resulted in death. Comorbidities, described in the second part of the MCCOD, are significant morbid conditions that contributed to death, but were not directly part of the causal chain that culminated in death^13^. Currently, microdata regarding deaths that occurred in Brazil between 1980 and 2019 are available, but due to the covid-19 pandemic, preliminary data for 2020 were made available in advance in April 2021 and this is the information that is part of this study.

MCCOD were analyzed using the following codes of the tenth revision of the International Classification of Diseases (ICD-10)^14^: cancer (C00-C97), CVD (I00-I99), external causes (V, W, X, Y) and ill-defined (R00-R99). To classify covid-19 deaths, we used the guidelines of the Health Surveillance Department of the Ministry of Health to codify the causes of death in the midst of the pandemic, in which the code B34.2 was used for deaths whose underlying cause was covid-19 in Brazil^15^.

For all deaths occurring in 2020, we estimated the age-standardized rates by the direct method, considering the age groups (0 to 19 years; 20 to 29 years; 30 to 39 years; 40 to 49 years; 50 to 59 years; 60 to 69 years; 70 to 79 years and 80 years or older) and using as standard the Brazilian population projected for 2020 by the IBGE.

Then, we recorded the deaths from the two main causes nationally and regionally in 2020 (cancer and CVD) and the deaths from covid-19. Correction factors were applied to these deaths by proportional redistribution of those reported as ill-defined causes, considering the age groups already described and the geographical locations, according to the method proposed by Mathers et al.^16^ Deaths from ill-defined causes were 5.55% and 6.44% of the total deaths recorded in 2019 and 2020, respectively. Among the Brazilian regions, this proportion ranged from 2.97% (Midwest) to 8.20% (North) in 2019 and from 4.68% (South) to 8.31% (North) in 2020. The largest increases were observed in the Midwest (from 2.97% in 2019 to 5.09% in 2020) and South (from 3.52% in 2019 to 4.48% in 2020).

To estimate age-standardized rates, deaths without information on age or date of birth were excluded: n = 2,358 (0.17%) in 2019 and n = 30,893 (1.98%) in 2020.

We then estimated the expected deaths by underlying cause of death (cancer or CVD) per month and for the period between March and December 2020, in addition to estimating the expected deaths with these diseases as comorbidities for the same period of pandemic in 2020. For this estimate, we applied the mortality coefficients by age groups (0 to 19 years; 20 to 29 years; 30 to 39 years; 40 to 49 years; 50 to 59 years; 60 to 69 years; 70 to 79 years and 80 years or older) for cause, period and region in 2019 as standard to the population projected for 2020, in the same age groups and regions. In this estimate, deaths with no information about age for the disease categories under study were excluded (2019: n = 246; 0.04%; 2020: n = 201; 0.02%).

We estimated the difference between the number of deaths observed and the number of deaths expected in this period, and the percentage change of this difference.

The standardized mortality ratio (RMP) was estimated as the ratio between observed and expected deaths in the period. The intervals with 95% confidence for each RMP were estimated by using a Poisson distribution, as described by Breslow and Day^17^:


$$RMP_{I}=\frac{D\left(1-\frac{1}{9D}\right)-{\left(\frac{1.96}{3\sqrt{D}}\right)}^{3}}{E}$$

$$RMP_{s}=\frac{(D+1)\left(1-\frac{1}{9(D+1)}\right)-{\left(\frac{1.96}{3\sqrt{\left(D+1\right)}}\right)}^{3}}{E}$$

Where:

RMP_I_ is the lower limit for the 95% confidence interval of the SMR;

RMP_S_ is the upper limit for the 95% confidence interval of the SMR;

*D* is the number of deaths observed in 2020;

*E* is the number of deaths expected for 2020.

Finally, we estimated the percentages of deaths observed in 2020 and expected by comorbidity – based on the profile observed in 2019 – having as a total the sum of deaths classified as underlying cause and the ones classified as comorbidity. The ratio between these percentages was checked to assess whether or not this ratio increased.

All estimates were made using the statistical software Stata^18^.

## RESULTS

**Figure 1:**
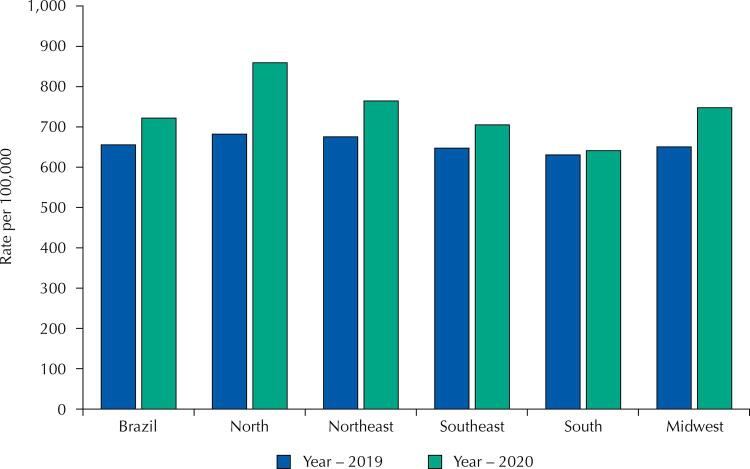
Distribution of mortality rates standardized by age for all causes of death in Brazil and Brazilian regions in 2019 and 2020.

In 2020, there were 1,560,088 deaths in Brazil. Compared to 2019, the general mortality rate standardized by age increased by 10.15%, going from 655.63 to 722.15 deaths per 100 thousand inhabitants. The largest increases were observed in the North (25.90%; from 682.11/100 thousand in 2019 to 858.79/100 thousand in 2020) and in the Northeast (12.99%; from 676.48/100 thousand in 2019 to 764.33/100 thousand in 2020) (Figure 1).

**Figure 2:**
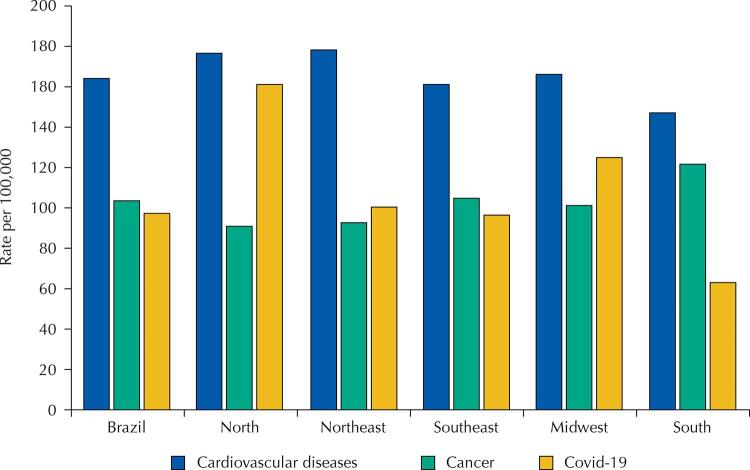
Distribution of mortality rates corrected and standardized by age for the three main causes of death in 2020 in Brazil and Brazilian regions.

In 2020, covid-19 was the third leading cause of death throughout Brazil and also in the Southeast and South regions, with an age-standardized rate of 97.58 per 100 thousand, second only to CVD (164.03/100 thousand) and cancer (103.60/100 thousand) and surpassing respiratory diseases (70.43/100 thousand). In the North (161.20/100 thousand), Northeast (100.50/100 thousand) and Midwest (125.21/100 thousand), covid-19 was the second only to CVD as a leading cause of death (Figure 2).

**Figure 3:**
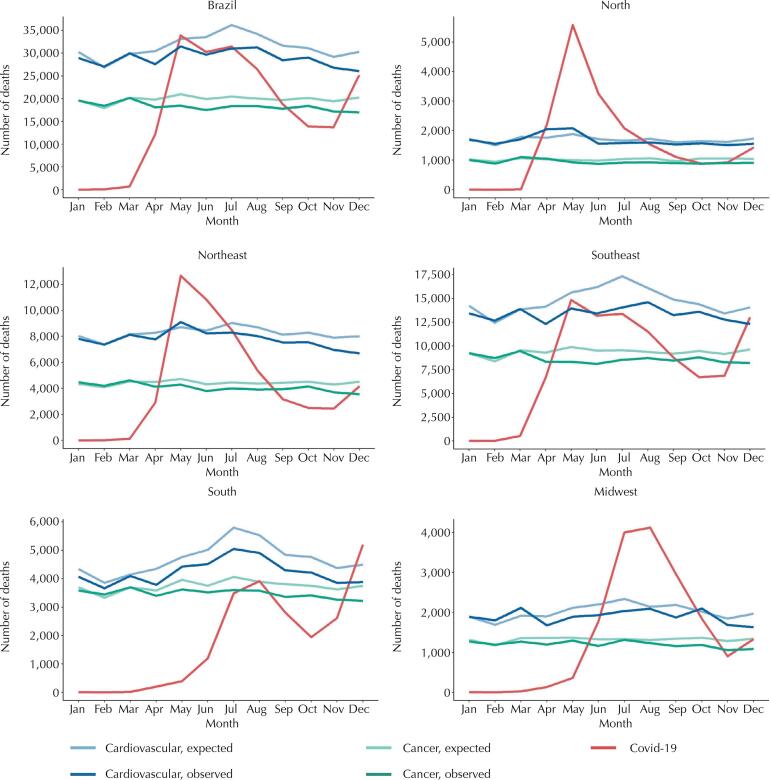
Observed and expected deaths from cancer, cardiovascular disease and covid-19 per month in 2020 in Brazil.

Figure 3 shows the observed and expected deaths from cancer and CVD, and deaths from covid-19 in the months of 2020 for Brazil and Brazilian regions. It is possible to note that, with the arrival of the covid-19 pandemic, starting in March, the observed deaths for both cancer and CVD declines in Brazil as a whole and in each region. In the North, however, the visual difference between the expected and observed death curves is not as striking as in the other regions.

In the period between March and December 2020, a total of 181,377 cancer deaths were reported in the SIM as underlying cause. This figure was 10% lower than expected (200,876), based on the mortality profile in the same period in 2019 (RMP = 0.90; 95%CI 0.90–0.91). This pattern repeated itself in all regions of the country where the SMR were always lower than the unit and statistically significant. The same finding among observed and expected deaths occurred for CVD in 2020 in the period, both for Brazil (291,375 and 319,561, respectively; SMR = 0.91; 95%CI 0.91–0.920) and for all regions (Table 1).

**Table 1:** Number of observed and expected deaths from cancer and cardiovascular diseases and standardized mortality ratio (SMR) in Brazil and Brazilian regions, between March and December 2020.

Underlying cause	Region	Observed deaths	Expected deaths	SMR (95%CI)	Difference (n)	Variation (%)
**Cancer**	**North**	9,416	10,333	0.91 (0.89–0.93)	-917	-8.87
	**Northeast**	40,047	44,645	0.90 (0.89–0.91)	-4,598	-10.30
	**Southeast**	85,302	94,640	0.90 (0.90–0.91)	-9,338	-9.87
	**South**	34,682	37,878	0.92 (0.91–0.93)	-3,196	-8.44
	**Midwest**	11,930	13,394	0.89 (0.87–0.91)	-1,464	-10.93
	**Brazil**	181,377	200,876	0.90 (0.90–0.91)	-19,499	-9.71
**Cardiovascular diseases**	**North**	16,753	17,132	0.98 (0.96–0.99)	-379	-2.21
	**Northeast**	78,324	83,630	0.94 (0.93–0.94)	-5,306	-6.34
	**Southeast**	134,239	150,050	0.89 (0.89–0.90)	-15,811	-10.54
	**South**	43,028	48,082	0.89 (0.89–0.90)	-5,054	-10.51
	**Midwest**	19,031	20,621	0.92 (0.91–0.94)	-1,590	-7.71
	**Brazil**	291,375	319,561	0.91 (0.91–0.92)	-28,186	-8.82

By analyzing the selected comorbidities separately, an opposite pattern was identified, with an increase in deaths observed for cancer, leading to an SMR of 1.82 (95%CI 1.79–1.85). A higher number of deaths observed than expected within this category was detected in all regions, especially in the North, where the SMR was 2.55 (95%CI 2.37–2.73). Regarding CVD as comorbidity, excess deaths were also found in Brazil as a whole (RMP = 1.78; 95%CI 1.77–1.79) and by regions. The highest SMR was also seen in the North (SMR = 2.46; 95%CI 2.41–2.50) (Table 2).

**Table 2:** Number of observed and expected deaths from cancer and cardiovascular diseases as comorbidity and standardized mortality ratio (SMR) in Brazil and Brazilian regions between March and December 2020.

Comorbidity	Region	Observed deaths	Expected deaths	SMR (95%CI)	Difference (n)	Variation (%)
**Cancer**	**North**	744	292	2.55 (2.37–2.73)	452	154.87
	**Northeast**	3,776	2,026	1.86 (1.80–1.92)	1,750	86.41
	**Southeast**	9,928	5,399	1.84 (1.80–1.87)	4,529	83.87
	**South**	2,487	1,561	1.59 (1.53–1.66)	926	59.34
	**Midwest**	1,196	683	1.75 (1.65–1.85)	513	75.09
	**Brazil**	**18,131**	**9,959**	**1.82 (1.79–1.85)**	**8,172**	**82.05**
**Cardiovascular diseases**	**North**	11,651	4,738	2.46 (2.41–2.50)	6,913	145.90
	**Northeast**	45,298	23,476	1.93 (1.91–1.95)	21,822	92.96
	**Southeast**	91,923	51,876	1.77 (1.76–1.78)	40,047	77.20
	**South**	25,177	17,832	1.41 (1.39–1.43)	7,345	41.19
	**Midwest**	14,155	7,899	1.79 (1.76–1.82)	6,256	79.20
	**Brazil**	**188,204**	**105,791**	**1.78 (1.77–1.79)**	**82,413**	**77.90**

## DISCUSSION

Covering the entire period of the pandemic in 2020 and using SIM data from all over Brazil, we were able to identify a decline of almost 10% in expected mortality from cancer and CVD, compared to 2019. This drop pattern occurred in the five regions of the country, with a slightly greater variation for CVD between the regions.

A possible explanation for the decline in mortality observed for the two groups of diseases studied here is the action of covid-19 as a competitive cause of death, resulting in migration of the underlying cause of death. Thus, prevalent cases of cancer and CVD, which would have a higher risk of death from these diseases, ended up having their deaths anticipated due to covid-19^19^. A study conducted in China between January and June 2020, covering three stages of the pandemic (first wave, second wave and recovery), showed a deficit in deaths from all causes, but did not present a statistically significant difference in cancer deaths and found excess in deaths from CVD^20^. Similar findings, without statistical significance, were reported in the first half of 2020 in the city of São Paulo for cancer (SMR = 0.9; 95%CI 0.67–1.20) and CVD (SMR = 0.9; 95%CI 0.68–1.15)^21^.

The competition for hospital beds and emergency care, as well as health professionals becoming ill, could have led to an increase in mortality from the chronic diseases studied. This would be expected particularly for acute manifestations of CVD, such as acute myocardial infarction (AMI), stroke and dissecting aortic aneurysm. Choudhary et al.^22^ showed that treatment of severe cardiac emergencies decreased during the pandemic and that, among the cases of acute coronary syndrome, the proportion of patients treated with conservative therapy increased. In the US, in the early months of the pandemic there were fewer hospital admissions for conditions requiring emergency treatment, mainly AMI, stroke and heart failure^23^.

The reduction in mortality in Brazil from both cancer and CVD in 2020 may not continue in the coming years. A greater difficulty in the monitoring and control of chronic conditions such as systemic hypertension and diabetes mellitus should have a later impact on the increase in mortality in the coming years, and actions to mitigate this effect have been recommended, such as increased use of telemedicine^24,25^.

In cancer patients, postponing surgeries to remove tumors and delaying chemo or radiotherapy treatment can cause the disease to progress, decreasing the chances of cure^26^. In Brazil, the pandemic caused the need for adaptations in early detection recommendations in order to prioritize the investigation of cases with suspicious signs and symptoms^27^. The decrease in these actions perceived in 2020^28^ is worrying because it implies a future impact on cancer mortality, particularly in low and middle income countries^29^.

Some studies have focused on estimating, through modeling, the future impact of the Covid-19 pandemic on mortality due to delayed diagnosis^30^. In the UK, it has been estimated that in the next five years there will be 15.3 to 16.6% of additional deaths from colorectal cancer and 7.9 to 9.6% from breast cancer, 5.8 to 6.0% from esophageal cancer and 4.8 to 5.3% from lung cancer^30^. In France, excess cancer mortality was estimated from one thousand to six thousand patients in the coming years^31^. In Australia, a significant impact is expected in terms of additional deaths and health costs due to changes in the stage of the disease at the beginning of treatment in cases of breast, lung, colorectal cancer (from stage I to II) and melanoma (from stage T1 to T2)^32^. In India, excess mortality related to cervical cancer is expected^33^.

The pandemic has been regarded by some as an opportunity to reduce the unnecessary and harmful use of health services, serving as a natural experiment in reducing the harms arising from these practices^34^. It is possible that overdiagnosis and overtreatment, resulting from screening without indication, especially for prostate and breast cancer, have decreased during the pandemic, especially because both are very prevalent practices in Brazil^35,36^. Although there is evidence of increased CVD mortality associated with overdiagnosis and overtreatment of cancer, and especially breast cancer, it is unlikely that a possible reduction has impacted the results presented here^37^.

The differences observed between the Brazilian regions are a reflection of the inequalities of access to health prior to the covid-19 pandemic^38^. As in other parts of the world, with the pandemic these inequalities have been amplified^39^, compromising the necessary measures to control the spread of the virus and highlighting the difficulties of access to health services. The North and Northeast regions have the lowest rates of human development in the country, the highest rates of inequality and the worst access to health services^38^. In addition, it is thought that the P1 variant emerged in the North^40^, possibly helping to increase cases and deaths at the end of 2020 because it is more transmissible^41^.

In the midst of the pandemic, these factors add to the already high load of NCDs in the North and Northeast regions, described since 2008 as resulting from the inequalities mentioned here^42^. Unlike the other regions of Brazil, the trend of mortality from CVD^43^ and cancer^44^ in these regions in years prior to the pandemic was upward, which may have led, especially in the North, to the largest excesses of deaths seen of these diseases as comorbidity during the pandemic.

In contrast to the fall in mortality of the diseases studied here as the underlying cause of death in Brazil, we observed a significant increase in mortality from cancer and CVD as comorbidities, respectively 82.1% and 77.9%. This increase ranged from 59.3% in the South to 154.9% in the North for cancer and from 41.2% in the South to 145.9% in the North for CVD.

The rules for reporting causes of death in the SIM are well established for cancer^45^ which, is generally recorded as an underlying cause. In the case of CVD, the probability of being reported as comorbidity at death is higher than in the case of cancer. With the pandemic, Brazil followed the international standard proposed by the WHO for certifying causes of death applied to the SIM^46^, which indicates that comorbidities such as cancer should not be considered an undelying cause of death even if they triggered a severe course of covid-19; instead, they should be record in the second part of the MCCOD.

The findings of this study reinforce an important concern in the pandemic about cancer as a comorbidity of patients with covid-19, namely that cancer can affect the immune system, making cancer patients more susceptible to infections, whether by the immunosuppressive effect of chemotherapy and/or by the impairment of lung capacity, more common in patients with lung cancer.

CVD are recognized as one of the main comorbidities associated with a worse prognosis in covid-19 patients^47^ and they may also be involved as antecedent causes in the causal chain of death of patients with covid-19, an aspect not investigated in this study. Cardiac patients infected with SARS-CoV-2 have shown a higher risk of morbidity and mortality. The pathophysiological mechanism of SARS caused by this virus is characterized by an overproduction of inflammatory cytokines that lead to systemic inflammation and multiple organ dysfunction that acutely affects the cardiovascular system^48,49^. Myocarditis is a major complication in covid-19^50^. Of the patients with covid-19, 7% have myocardial injury resulting from the infection, a number that rises to 22% in severe patients^51^. Disorders in angiotensin converting enzyme (ACE) receptors also play an important role in pathogenesis, leading to cardiomyopathy and heart failure^50^.

Systemic arterial hypertension, arrhythmias, cardiomyopathies and coronary artery disease are among the main comorbidities in patients with severe covid-19^50^. A study that analyzed the role of CVD as comorbidity and a poor prognostic factor with data from SIVEP-Gripe in patients aged 20 years or older, hospitalized with covid-19, confirmed by quantitative RT-PCR, until August 2020, showed that 84% of them had one or more comorbidities, including the following types of diseases: cardiovascular, renal, neurological, hematological, hepatic, diabetes, chronic respiratory diseases, obesity or immunosuppression. These patients presented higher mortality compared to those without comorbidity^52^.

The limitation of this study is the fact that the SIM data for 2020 is still preliminary and it is possible that the frequencies change after the definitive consolidation. As a result of the covid-19 pandemic, the Ministry of Health anticipated the release of the preliminary database, provided for by the regulations for the period between June 30 and August 30, 2021^53^. Previous studies on the impact of covid-19 on mortality in Brazil in 2020 used the Civil Registry database^4^ or the respiratory syndrome notification database, SIVEP-Gripe^52,54^. These bases, however, do not allow to perform the analyzes presented here because they do not provide detailed information about underlying causes of death or comorbidities. For this reason, we chose to use data from the SIM for 2020, even if they are preliminary. In fact, we observed on this preliminary database that some fields, such as age or birth date, had a greater amount of ignored data. The percentage of MCCOD without this information was 0.17% in 2019, and in 2020 it rose to 1.98%. This amount, however, is small and should not significantly change the findings of this study. Similarly, the increase in deaths from ill-defined causes that after redistribution may have helped to attenuate the negative difference between expected and observed deaths from cancer and CVD in Brazil and Brazilian regions. The increase in ignored data and ill-defined causes in the period is a reflection of the health crisis, resulting from difficulties in accessing health services and difficulties related to the recording of information.

When considering the sum of deaths due to underlying cause and comorbidity, the proportion of deaths where each of them appears as comorbidity is lower than the proportion as underlying cause for CVD, and even lower for cancer. In 2020, however, there was a clear increase in the report of these two groups of death as comorbidity. For cancer, 4.7% was expected and 9.1% was observed, an increase of 92%. Among CVD, there was also an increase between expected and observed (24.9% to 39.2%, respectively) (data not presented). This situation reinforces the idea that covid-19 must have been responsible for a significant part of deaths in patients with cancer and CVD. The pandemic implied definitions in the chain of events rule related to death from covid-19, which may also have influenced the increase in the number of cancer records as comorbidity.

## CONCLUSIONS

The results presented here provide a preliminary analysis on the changes in the mortality pattern as a result of the pandemic. The increased number of deaths cited as comorbidities for both cancer and CVD deserves special attention and indicates the need to monitor the impact of covid-19 among patients with these morbidities. We should highlight the observation of a pattern of increased CVD as comorbidities in the North during the pandemic, which may be the result of a joint effect of the high burden of these diseases and lower access to health care. These analyses are important components to evaluate and guide health interventions and policies aimed at controlling deaths directly or indirectly associated with covid-19.

## Footnotes

a 2019 data available at: ftp://ftp.datasus.gov.br/dissemin/publicos/SIM/CID10/DORES; 2020 preliminary data available at ftp://ftp.datasus.gov.br/dissemin/publicos/SIM/PRELIM/DORES; accessible via Internet Explorer or another application for FTP access. Last accessed on February 18, 2022.

b Instituto Brasileiro de Geografia e Estatística, Sistema IBGE de Recuperação Automática -SIDRA. Projeções da População: projeção da população - edição 2018: tabelas. Rio de Janeiro: IBGE; 2018 [cited 2021 Apr 29]. Available from: https://sidra.ibge.gov.br/pesquisa/projecao-da-populacao/tabelas

## References

[1] Islam N, Shkolnikov VM, Acosta RJ, Klimkin I, Kawachi I, Irizarry RA, et al. Excess deaths associated with covid-19 pandemic in 2020: age and sex disaggregated time series analysis in 29 high income countries. BMJ. 2021;373:n1137. 10.1136/bmj.n1137 PMC8132017 34011491

[2] Candido DS, Claro IM, Jesus JG, Souza WM, Moreira FRR, Dellicour S, et al. Evolution and epidemic spread of SARS-CoV-2 in Brazil. Science. 2020;369(6508):1255-60. 10.1126/science.abd2161 PMC7402630 32703910

[3] França EB, Ishitani LH, Teixeira RA, Abreu DMX, Corrêa PRL, Marinho F, et al. Óbitos por COVID-19 no Brasil: quantos e quais estamos identificando? Rev Bras Epidemiol. 2020;23:E200053. 10.1590/1980-549720200053 32578810

[4] Azevedo e Silva G, Jardim BC, Santos CVB. Excesso de mortalidade no Brasil em tempos de COVID-19. Cienc Saude Coletiva. 2020;25(9):3345-54. 10.1590/1413-81232020259.23642020 32876246

[5] Orellana JDY, Cunha GM, Marrero L, Horta BL, Leite IC, Orellana JDY, et al. Explosão da mortalidade no epicentro amazônico da epidemia de COVID-19. Cad Saude Publica. 2020;36(7):e00120020 10.1590/0102-311x00120020 32638881

[6] Andrade CLT, Pereira CCA, Martins M, Lima SML, Portela MC. COVID-19 hospitalizations in Brazil’s Unified Health System (SUS). PLoS One. 2020;15(12):e0243126. 10.1371/journal.pone.0243126 PMC7728222 33301479

[7] The GBD 2019 Diseases and Injuries Collaborators. Global burden of 369 diseases and injuries in 204 countries and territories, 1990-2019: a systematic analysis for the Global Burden of Disease Study 2019. Lancet. 2020;396(10258):1204-22. 10.1016/S0140-6736(20)30925-9 PMC7567026 33069326

[8] Izcovich A, Ragusa MA, Tortosa F, Lavena Marzio MA, Agnoletti C, Bengolea A, et al. Prognostic factors for severity and mortality in patients infected with COVID-19: a systematic review. PLoS One. 2020;15(11):e0241955. 10.1371/journal.pone.0241955 PMC7671522 33201896

[9] Ng WH, Tipih T, Makoah NA, Vermeulen JG, Goedhals D, Sempa JB, et al. Comorbidities in SARS-CoV-2 patients: a systematic review and meta-analysis. MBio. 2021;12(1):e03647-20. 10.1128/mBio.03647-20 PMC7885108 33563817

[10] 0. Panamerican Health Organization. Considerations for the reorganization of cancer services during the COVID-19 pandemic. Washington, DC: PAHO: 2020 [cited 2021 Jun 6]. Available from: https://iris.paho.org/bitstream/handle/10665.2/52263/PAHOEIHHACOVID-19200004_eng.pdf

[11] Asokan I, Rabadia SV, Yang EH. The COVID-19 pandemic and its impact on the cardio-oncology population. Curr Oncol Rep. 2020;22(6):60. 10.1007/s11912-020-00945-4 PMC7253235 32462289

[12] Ferlay J, Soerjomataram I, Dikshit R, Eser S, Mathers C, Rebelo M, et al. Cancer incidence and mortality worldwide: sources, methods and major patterns in GLOBOCAN 2012. Int J Cancer. 2015;136(5):E359-86. 10.1002/ijc.29210 25220842

[13] Ministério da Saúde (BR), Fundação Nacional de Saúde. Manual de instruções para o preenchimento da declaração de óbito. Brasília, DF; 2011. (Série A. Normas e Manuais Técnicos).

[14] World Health Organization. International Statistical Classification of Diseases and Related Health Problems. 10th revision. Geneva (CH): WHO; 2019 [cited 2021 May 2]. Available from: https://icd.who.int/browse10/2019/en#/

[15] Ministério da Saúde (BR), Secretaria de Vigilância em Saúde, Departamento de Análise em Saúde e Vigilância de Doenças não Transmissíveis. Orientações para codificação das causas de morte no contexto da COVID-19. Brasília, DF; 2020.

[16] Mathers CD, Bernard C, Iburg KM, Inoue M, Fat DM, Shibuya K, et al. Global burden of disease in 2002: data sources, methods and results, results. Geneva (CH): WHO; 2003 [cited 2021 Jun 6]. (Global Programme on Evidence for Health Policy Discuss Paper; nº 54). Available from: https://www.who.int/healthinfo/paper54.pdf

[17] Breslow NE, Day NE. Statistical methods in cancer research. Vol. 2: The design and analysis of cohort studies. Lyon (FR): IARC; 1987. p. 69-71. (IARC Scientific Publication; nº 82). 3329634

[18] StataCorp. Stata Statistical Software: Release 17. College Station, TX: StataCorp LP; 2021.

[19] Institute for Health Metrics and Evaluations. Estimation of total mortality due to COVID-19. Seattle, WA: IHME; 2021 [cited 2021 Jun 6]. Available from: http://www.healthdata.org/special-analysis/estimation-excess-mortality-due-covid-19-and-scalars-reported-covid-19-deaths

[20] 0. Li L, Hang D, Dong H, Yuan-Yuan C, Bo-Heng L, Ze-Lin Y, et al. Temporal dynamic in the impact of COVID−19 outbreak on cause-specific mortality in Guangzhou, China. BMC Public Health. 2021;21(1):883. 10.1186/s12889-021-10771-3 PMC8105693 33964914

[21] Fernandes GA, Nassar Junior AP, Azevedo e Silva G, Feriani D, Silva ILAF, Caruso P, et al. Excess mortality by specific causes of deaths in the city of São Paulo, Brazil, during the COVID-19 pandemic. PLoS One. 2021;16(6):e0252238. 10.1371/journal.pone.0252238 PMC8184000 34097694

[22] Choudhary R, Gautam D, Mathur R, Choudhary D. Management of cardiovascular emergencies during the COVID-19 pandemic. Emerg Med J. 2020;37(12):778-80. 10.1136/emermed-2020-210231 33051275

[23] Baum A, Schwartz MD. Admissions to Veterans Affairs hospitals for emergency conditions during the COVID-19 pandemic. JAMA. 2020;324(1):96-9. 10.1001/jama.2020.9972 PMC7275263 32501493

[24] Gujral UP, Johnson L, Nielsen J, Vellanki P, Haw JS, Davis GM, et al. Preparedness cycle to address transitions in diabetes care during the COVID-19 pandemic and future outbreaks. BMJ Open Diabetes Res Care. 2020;8(1):e001520. 10.1136/bmjdrc-2020-001520 PMC7385737 32690631

[25] Malta DC, Gomes CS, Barros MBA, Lima MG, Almeida WS, Sá ACMGN, et al. Doenças crônicas não transmissíveis e mudanças nos estilos de vida durante a pandemia de COVID-19 no Brasil. Rev Bras Epidemiol. 2021;24:E210009. 10.1590/1980-549720210009 33950138

[26] Riera R, Bagattini AM, Pacheco RL, Pachito DV, Roitberg F, Ilbawi A. Delays and disruptions in cancer health care due to COVID-19 pandemic: systematic review. JCO Glob Oncol. 2021;7:311-23. 10.1200/GO.20.00639 PMC8081532 33617304

[27] Migowski A, Corrêa FM. Recomendações para detecção precoce de câncer durante a pandemia de covid-19 em 2021. Rev APS. 2020;23(1):235-40.

[28] Patt D, Gordan L, Diaz M, Okon T, Grady L, Harmison M, et al. Impact of COVID-19 on cancer care: how the pandemic is delaying cancer diagnosis and treatment for American seniors. JCO Clin Cancer Inform. 2020;4:1059-71. 10.1200/cci.20.00134 PMC7713534 33253013

[29] Villain P, Carvalho AL, Lucas E, Mosquera I, Zhang L, Muwonge R, et al. Cross-sectional survey of the impact of the COVID-19 pandemic on cancer screening programs in selected low- and middle-income countries: study from the IARC COVID-19 impact study group. Int J Cancer. 2021;149(1):97-107. 10.1002/ijc.33500 PMC8014228 33533501

[30] 0. Maringe C, Spicer J, Morris M, Purushotham A, Nolte E, Sullivan R, et al. The impact of the COVID-19 pandemic on cancer deaths due to delays in diagnosis in England, UK: a national, population-based, modelling study. Lancet Oncol. 2020;21(8):1023-34. 10.1016/S1470-2045(20)30388-0 PMC7417808 32702310

[31] Blay JY, Boucher S, Le Vu B, Cropet C, Chabaud S, Perol D, et al. Delayed care for patients with newly diagnosed cancer due to COVID-19 and estimated impact on cancer mortality in France. ESMO Open. 2021;6(3):100134. 10.1016/j.esmoop.2021.100134 PMC8134718 33984676

[32] Degeling K, Baxter NN, Emery J, Jenkins MA, Franchini F, Gibbs P, et al. An inverse stage-shift model to estimate the excess mortality and health economic impact of delayed access to cancer services due to the COVID-19 pandemic. Asia Pac J Clin Oncol. 2021;17(4):359-67. 10.1111/ajco.13505 PMC8014813 33567163

[33] Gupta N, Chauhan AS, Prinja S, Pandey AK. Impact of COVID-19 on outcomes for patients with cervical cancer in India. JCO Glob Oncol. 2021;7:716-25. 10.1200/go.20.00654 PMC8162960 33999708

[34] Moynihan R, Johansson M, Maybee A, Lang E, Légaré F. Covid-19: an opportunity to reduce unnecessary healthcare. BMJ. 2020;370:m2752. 10.1136/bmj.m2752 32665257

[35] Soares SCM, Cancela MC, Migowski A, Souza DLB. Digital rectal examination and its associated factors in the early detection of prostate cancer: a cross-sectional population-based study. BMC Public Health. 2019;19(1):1573. 10.1186/s12889-019-7946-z PMC6881979 31775710

[36] Migowski A, Dias MBK, Nadanovsky P, Azevedo e Silva G, Sant’Ana DR, Stein AT. Guidelines for early detection of breast cancer in Brazil. III – Challenges for implementation. Cad Saude Publica. 2018;34(6):e00046317. 10.1590/0102-311X00046317 29952397

[37] Migowski A, Nadanovsky P, Vianna CMM. Mortalidade cardiovascular associada ao rastreamento mamográfico. Rev Bras Cancerol. 2019;65(3):e-02335. 10.32635/2176-9745.RBC.2019v65n3.335

[38] Dantas MNP, Souza DLB, Souza AMG, Aiquoc KM, Souza TA, Barbosa IR. Fatores associados ao acesso precário aos serviços de saúde no Brasil. Rev Bras Epidemiol. 2021;24:E210004. 10.1590/1980-549720210004 33331413

[39] Marmot M, Allen J. COVID-19: exposing and amplifying inequalities. J Epidemiol Community Health. 2020;74(9):681-2. 10.1136/jech-2020-214720 PMC7577092 32669357

[40] 0. Fujino T, Nomoto H, Kutsuna S, Ujiie M, Suzuki T, Sato R, et al. Novel SARS-CoV-2 variant in travelers from Brazil to Japan. Emerg Infect Dis. 2021;27(4):1243-5. 10.3201/eid2704.210138 PMC8007308 33567247

[41] Kerr LRFS, Kendall C, Almeida RLF, Ichihara MY, Aquino EML, Silva AAM, et al. COVID-19 in northeast Brazil: first year of the pandemic and uncertainties to come. Rev Saude Publica. 2021;55:35. 10.11606/s1518-8787.2021055003728 PMC8139846 34105604

[42] Leite IC, Valente JG, Schramm JMA, Daumas RP, Rodrigues RN, Santos MF, et al. Carga de doença no Brasil e suas regiões, 2008. Cad Saude Publica. 2015;31(7):1551-64. 10.1590/0102-311X00111614 26248109

[43] Baptista EA, Queiroz BL. The relation between cardiovascular mortality and development: a study of small areas in Brazil, 2001-2015. Demogr Res. 2019;41(51):1437-52. 10.4054/DemRes.2019.41.51

[44] Azevedo e Silva G, Jardim BC, Ferreira VM, Junger WL, Girianelli VR. Mortalidade por câncer nas capitais e no interior do Brasil: uma análise de quatro décadas. Rev Saude Publica. 2020;54:126. 10.11606/s1518-8787.2020054002255 PMC7688260 33295593

[45] Ministério da Saúde (BR), Secretaria de Vigilância em Saúde, Coordenação Geral de Informação e Análise Epidemiológica. Protocolos de codificações especiais em mortalidade. Brasília, DF; 2013.

[46] World Health Organization. International guidelines for certification and classification (coding) of COVID-19 as cause of death. Based on ICD International Statistical Classification of Diseases. Geneva (CH): WHO; 2020.

[47] Nandy K, Salunke A, Pathak SK, Pandey A, Doctor C, Puj K, et al. Coronavirus disease (COVID-19): a systematic review and meta-analysis to evaluate the impact of various comorbidities on serious events. Diabetes Metab Syndr. 2020;14(5):1017-25. 10.1016/j.dsx.2020.06.064 PMC7331565 32634716

[48] Azevedo RB, Botelho BG, Hollanda JVG, Ferreira LVL, Andrade LZJ, Oei SSML, et al. Covid-19 and the cardiovascular system: a comprehensive review. J Hum Hypertens. 2021;35:4-11. 10.1038/s41371-020-0387-4 PMC7384729 32719447

[49] Aleksova A, Gagno G, Sinagra G, Beltrami AP, Janjusevic M, Ippolito G, et al. Effects of SARS-CoV-2 on cardiovascular system: the dual role of angiotensin converting enzyme 2 (ACE2) as the virus receptor and homeostasis regulator review. Int J Mol Sci. 2021;22(9):4526. 10.3390/ijms22094526 PMC8123609 33926110

[50] 0. Babapoor-Farrokhran S, Gill D, Walker J, Rasekhi RT, Bozorgnia B, Amanullah A. Myocardial injury and COVID-19: possible mechanisms. Life Sci. 2020;253:117723. 10.1016/j.lfs.2020.117723 PMC7194533 32360126

[51] Clerkin KJ, Fried JA, Raikhelkar J, Sayer G, Griffin JM, Masoumi A, et al. COVID-19 and cardiovascular disease. Circulation. 2020;141(20):1648-55. 10.1161/CIRCULATIONAHA.120.046941 32200663

[52] Ranzani OT, Bastos LSL, Gelli JGM, Marchesi JF, Baião F, Hamacher S, et al. Characterisation of the first 250 000 hospital admissions for COVID-19 in Brazil: a retrospective analysis of nationwide data. Lancet Respir Med. 2021;9(4):407-18. 10.1016/S2213-2600(20)30560-9 PMC7834889 33460571

[53] Ministerio da Saúde (BR), Secretaria de Vigilância em Saúde. Portaria N^o^ 116, de 11 de fevereiro de 2009. Regulamenta a coleta de dados, fluxo e periodicidade de envio das informações sobre óbitos e nascidos vivos para os Sistemas de Informações em Saúde sob gestão da Secretaria de Vigilância em Saúde. Brasília, DF; 2009.

[54] Azevedo e Silva G, Jardim BC, Lotufo PA. Mortalidade por COVID-19 padronizada por idade nas capitais das diferentes regiões do Brasil. Cad Saude Publica. 2021;37(6):e00039221. 10.1590/0102-311x00039221 34259712

