# Supplementary material for: Covid-19 in Brazil in 2020: impact on deaths from cancer and cardiovascular diseases
Source: Rev Saude Publica. 2022 Apr 11;56:22. doi: 10.11606/s1518-8787.2022056004040 (PMC9004704; doi:10.11606/s1518-8787.2022056004040)
Supplement: Translation

# Translation: Covid-19 no Brasil em 2020: impacto nas mortes por câncer e doenças cardiovasculares

**Authors:** Beatriz Cordeiro Jardim, Arn Migowski, Flávia de Miranda Corrêa, Gulnar Azevedo e Silva

**Affiliations:** I Brasil Instituto Nacional de Câncer. Coordenação de Prevenção e Vigilância. Divisão de Detecção Precoce e Apoio à Organização de Rede. Rio de Janeiro, RJ, Brasil; II Brasil Universidade do Estado do Rio de Janeiro. Instituto de Medicina Social Hésio Cordeiro. Rio de Janeiro, RJ, Brasil; III Brasil Instituto Nacional de Cardiologia. Coordenação de Ensino e Pesquisa. Núcleo de Epidemiologia. Rio de Janeiro, RJ, Brasil

**Keywords:** Covid-19, Doenças Cardiovasculares, Neoplasias, Comorbidade, Mortalidade

## Abstract

**OBJETIVO:**

Analisar o impacto da pandemia de covid-19 sobre a mortalidade por câncer e por doenças cardiovasculares (DCV) como causa básica e comorbidade no Brasil e em suas regiões em 2020.

**MÉTODOS:**

Foram utilizadas as bases de dados de 2019 e 2020 do Sistema de Informações de Mortalidade (SIM), analisando os óbitos ocorridos entre março e dezembro de cada ano que tiveram o câncer e as DCV como causa básica ou como comorbidade. Também foram analisados os óbitos por covid-19 em 2020. Para o cálculo da Razão de Mortalidade Padronizada (RMP) e estimativa do excesso de mortes, os dados de 2019 foram considerados como padrão.

**RESULTADOS:**

Entre março e dezembro de 2020 ocorreram no Brasil 181.377 mortes por câncer e 291.375 mortes por doenças cardiovasculares, indicando redução de 9,7% e de 8,8%, respectivamente, em relação ao mesmo período do ano anterior. O padrão foi mantido nas cinco regiões brasileiras, com menor variação para o câncer (-8,4% na Região Sul a -10,9% na Região Centro-Oeste). Para as DCV houve uma maior variação, de -2,2% na Região Norte até -10,5 nas regiões Sudeste e Sul. No mesmo período de 2020, essas enfermidades foram classificadas como comorbidade em 18.133 óbitos por câncer e 188.204 óbitos por doenças cardiovasculares, indicando um excesso proporcional, se comparado aos dados de 2019, de 82,1% e 77,9%, respectivamente. Esse excesso foi mais expressivo na Região Norte, com razão de 2,5 entre mortes observadas e esperadas, para as duas condições estudadas.

**CONCLUSÕES:**

O excesso de óbitos por câncer e DCV como comorbidade em 2020 pode indicar que a covid-19 teve um importante impacto entre pacientes portadores dessas condições.

## INTRODUÇÃO

A pandemia de covid-19, doença causada pelo novo coronavírus (SARS-CoV-2), modificou de forma substancial o perfil de mortalidade em muitos países. Em 2020, ocorreram aproximadamente um milhão de mortes adicionais em 29 países de alta renda em comparação aos quatro anos anteriores^1^. No Brasil, vários autores mostraram que ao comparar com anos anteriores, em 2020 houve claramente um excesso de mortes^2^, bem como aumento no índice de mortalidade hospitalar^6^.

Historicamente, a partir da segunda metade do século passado, a carga das doenças crônicas não transmissíveis (DCNT) assumiu protagonismo, sendo responsável por mais de 60% dos óbitos em todo o mundo atualmente^7^. As doenças cardiovasculares (DCV) e o câncer são as duas principais causas de morte no Brasil há muitas décadas.

Evidências experimentais e observacionais mostram um risco consistentemente maior de evolução para caso grave de covid-19 em indivíduos idosos e com comorbidades^8,9^, por conta da síndrome respiratória aguda (SARS). Entre pacientes com DCNT, como o câncer e as DCV, as condições de saúde podem se agravar em decorrência direta ou indireta da pandemia, seja por maior fragilidade ou pior resposta à infecção pelo coronavírus de tipo 2, causador da SARS (SARS-Cov-2), seja pelas dificuldades de acesso e continuidade do cuidado dessas condições crônicas nos serviços de saúde ou por prejuízo às ações de detecção precoce, confirmação diagnóstica e tratamento inicial^10^. Consequentemente, avaliar a morbimortalidade em indivíduos com risco aumentado para o desenvolvimento de covid-19 grave é imprescindível para determinar estratégias preventivas e terapêuticas para esse grupo específico, bem como investigar o impacto da pandemia em suas doenças de base^11^.

A análise das informações acessíveis no Sistema Único de Saúde (SUS) é essencial para avaliar como a covid-19 tem modificado o perfil de mortalidade do país, assim como para identificar situações que visem o aprimoramento da vigilância em saúde. O objetivo deste estudo foi analisar o impacto da pandemia de covid-19 na mortalidade por câncer e DCV, tanto como causa básica quanto como comorbidade no Brasil e em suas regiões em 2020.

## MÉTODOS

Os dados sobre óbitos ocorridos em 2019 e em 2020 (base preliminar disponibilizada) utilizados neste estudo foram obtidos do Sistema de Informação sobre Mortalidade (SIM), no portal do Departamento de Informática do SUS (Datasus)^a^. Já as informações populacionais geral e por região foram obtidas das projeções populacionais do Instituto Brasileiro de Geografia e Estatística (IBGE), edição de 2018^b^.

O SIM tem sido aperfeiçoado desde sua criação, em 1975, e sua qualidade e cobertura são consideras boas dentro dos padrões internacionais^12^. Os dados das declarações de óbito (DO) que alimentam o SIM são preenchidos por médicos e classificados seguindo regras que permitem estabelecer a causa básica da morte, as causas antecedentes e as contribuintes (ou comorbidades). O modelo único de DO adotado no Brasil classifica as causas de morte conforme padrão da Organização Mundial da Saúde (OMS). Seguindo a cadeia causal de morte, a causa básica é a doença ou lesão (ou circunstâncias no caso de causas externas) que iniciou a cadeia de acontecimentos patológicos expressos nas causas antecedentes e que resultaram diretamente na morte. Já as comorbidades, descritas na segunda parte da DO, são condições mórbidas significativas que contribuíram para a morte, mas que não fizeram parte diretamente da cadeia causal que culminou com o óbito^13^. Atualmente, os microdados referentes aos óbitos ocorridos no Brasil entre 1980 e 2019 encontram-se disponíveis, sendo que, em função da pandemia de covid-19, os dados preliminares de 2020 foram disponibilizados antecipadamente em abril de 2021 e são essas informações que integram este estudo.

As declarações de óbito foram analisadas utilizando os seguintes códigos da Décima Revisão da Classificação Internacional de Doenças (CID-10)^14^: câncer (C00-C97), DCV (I00-I99), causas externas (V, W, X, Y) e mal definidas (R00-R99). Para classificar as mortes por covid-19 foram utilizadas as orientações da Secretaria de Vigilância em Saúde do Ministério da Saúde para codificação das causas de morte em meio à pandemia, em que o código B34.2 foi indicado para mortes cuja causa básica foi a covid-19 no Brasil^15^.

Para o conjunto dos óbitos ocorridos em 2020 foram calculadas taxas padronizadas por idade pelo método direto, considerando as faixas etárias (0 a 19 anos; 20 a 29 anos; 30 a 39 anos; 40 a 49 anos; 50 a 59 anos; 60 a 69 anos; 70 a 79 anos e 80 anos ou mais) e tendo como padrão a população brasileira projetada para 2020 pelo IBGE.

Posteriormente foram contabilizados os óbitos pelas duas principais causas nacional e regionalmente em 2020 (câncer e DCV) e os óbitos por covid-19. A esses óbitos foram aplicados fatores de correção por redistribuição proporcional daqueles classificados como causas mal definidas, considerando as faixas etárias já descritas e as localidades geográficas, de acordo com o método proposto por Mathers et al.^16^ Os óbitos por causas mal definidas corresponderam a 5,55% e 6,44% do total de óbitos registrados em 2019 e 2020, respectivamente. Entre as regiões brasileiras, essa proporção variou de 2,97% (Centro-Oeste) a 8,20% (Norte) em 2019 e de 4,68% (Sul) a 8,31% (Norte) em 2020. Os maiores incrementos foram observados nas Regiões Centro-Oeste (de 2,97% em 2019 para 5,09% em 2020) e Sul (de 3,52% em 2019 para 4,48% em 2020).

Para o cálculo das taxas padronizadas por idade foram excluídos os óbitos sem informação sobre idade ou data de nascimento: n = 2.358 (0,17%) em 2019 e n = 30.893 (1,98%) em 2020.

Em seguida calculou-se os óbitos esperados pelas causas básicas de morte (câncer ou DCV), por mês e para o período entre março e dezembro de 2020, além de calcular os óbitos esperados com essas doenças como comorbidades para o mesmo período de pandemia em 2020. Para esse cálculo, os coeficientes de mortalidade por faixas etárias (0 a 19 anos; 20 a 29 anos; 30 a 39 anos; 40 a 49 anos; 50 a 59 anos; 60 a 69 anos; 70 a 79 anos e 80 anos ou mais) por causa, período e localidade em 2019 foram aplicados como padrão à população projetada para 2020, nas mesmas faixas etárias e localidades. Nesse cálculo foram excluídos os óbitos sem informação sobre idade para as categorias de doenças em estudo (2019: n = 246; 0,04%; 2020: n = 201; 0,02%).

Foi calculada a diferença entre o número de óbitos observados e o número de óbitos esperados nesse período e a variação percentual dessa diferença.

A razão de mortalidade padronizada (RMP) foi calculada como sendo a razão entre os óbitos observados e os óbitos esperados no período. Os intervalos com 95% de confiança para cada RMP foram calculados assumindo uma distribuição Poisson, conforme descrito por Breslow e Day^17^:


$$RMP_{I}=\frac{D\left(1-\frac{1}{9D}\right)-{\left(\frac{1.96}{3\sqrt{D}}\right)}^{3}}{E}$$

$$RMP_{s}=\frac{(D+1)\left(1-\frac{1}{9(D+1)}\right)-{\left(\frac{1.96}{3\sqrt{\left(D+1\right)}}\right)}^{3}}{E}$$

Onde:

RMP_I_ é o limite inferior para o intervalo de confiança a 95% da RMP;

RMP_S_ é o limite superior para o intervalo de confiança a 95% da RMP;

*D* é o número de mortes observadas em 2020;

*E* é o número de mortes esperadas para 2020.

Por fim, foram calculados os percentuais de óbitos observados no ano de 2020 e esperados por comorbidade – com base no perfil verificado em 2019 – tendo como total a soma dos óbitos classificados como causa básica mais os classificados como comorbidade. A razão entre esses percentuais foi verificada para avaliar se houve ou não aumento dessa relação.

Todos os cálculos foram realizados no programa estatístico Stata^18^.

## RESULTADOS

No ano de 2020 ocorreram 1.560.088 mortes no Brasil. Comparando-se a 2019, a taxa de mortalidade geral padronizada por idade teve um incremento de 10,15%, passando de 655,63 para 722,15 mortes por 100 mil habitantes. Os maiores incrementos foram observados nas Regiões Norte (25,90%; de 682,11/100 mil em 2019 para 858,79/100 mil em 2020) e na Região Nordeste (12,99%; de 676,48/100 mil em 2019 para 764,33/100 mil em 2020) (Figura 1).

**Figura 1:** Distribuição das taxas de mortalidade padronizadas por idade para o conjunto das causas de morte no Brasil e regiões em 2019 e 2020.

Em 2020, a covid-19 foi a terceira principal causa de morte em todo o Brasil e também nas regiões Sudeste e Sul, com uma taxa padronizada por idade de 97,58 por 100 mil, posicionando-se após as DCV (164,03/100 mil) e câncer (103,60/100 mil), ultrapassando as doenças respiratórias (70,43/100 mil). Nas regiões Norte (161,20/100 mil), Nordeste (100,50/100 mil) e Centro-Oeste (125,21/100 mil) a covid-19 foi a segunda causa principal de morte, sendo superada apenas pelas DCV (Figura 2).

**Figura 2:** Distribuição das taxas de mortalidade corrigidas e padronizadas por idade para as três principais causas de morte em 2020 no Brasil e Regiões.

Na Figura 3 são apresentados os números de óbitos observados e esperados de câncer e de DCV, e os óbitos por covid-19 durante os meses de 2020 para o Brasil e regiões. É possível notar que, com a chegada da pandemia de covid-19, a partir do mês de março, há um recuo do número de óbitos observados tanto para câncer quanto para DCV no Brasil como um todo e em cada região. Na Região Norte, porém, a diferença visual entre as curvas de óbitos esperados e observados não é tão marcante quanto nas demais.

**Figura 3:** Mortes observadas e esperadas por câncer, doenças cardiovasculares e covid-19 por mês em 2020 no Brasil.

No período entre março e dezembro de 2020 foram classificados no SIM um total de 181.377 óbitos que tiveram como causa básica câncer. Esse número foi 10% menor do que o esperado (200.876), tomando-se como base o perfil de mortalidade no mesmo período em 2019 (RMP = 0,90; IC95% 0,90–0,91). Esse padrão se repetiu em todas as regiões do país onde as RMP foram sempre menores que a unidade e estatisticamente significativas. A mesma constatação entre os óbitos observados e esperados ocorreu para DCV em 2020 no período, tanto para o Brasil (291.375 e 319.561, respectivamente; RMP = 0,91; IC95% 0,91–0,920) quanto para todas as regiões (Tabela 1).

**Tabela 1:** Número de óbitos observados e esperados por câncer e por doenças cardiovasculares e razão de mortalidade padronizada (RMP) no Brasil e regiões, entre março e dezembro de 2020.

Causa básica	Região	Óbitos observados	Óbitos esperados	RMP (IC95%)	Diferença (n)	Variação (%)
**Câncer**	**Norte**	9.416	10.333	0,91 (0,89–0,93)	-917	-8,87
	**Nordeste**	40.047	44.645	0,90 (0,89–0,91)	-4.598	-10,30
	**Sudeste**	85.302	94.640	0,90 (0,90–0,91)	-9.338	-9,87
	**Sul**	34.682	37.878	0,92 (0,91–0,93)	-3.196	-8,44
	**Centro-Oeste**	11.930	13.394	0,89 (0,87–0,91)	-1.464	-10,93
	**Brasil**	**181.377**	**200.876**	**0,90 (0,90–0,91)**	**-19.499**	**-9,71**
**Doenças cardiovasculares**	**Norte**	16.753	17.132	0,98 (0,96–0,99)	-379	-2,21
	**Nordeste**	78.324	83.630	0,94 (0,93–0,94)	-5.306	-6,34
	**Sudeste**	134.239	150.050	0,89 (0,89–0,90)	-15.811	-10,54
	**Sul**	43.028	48.082	0,89 (0,89–0,90)	-5.054	-10,51
	**Centro-Oeste**	19.031	20.621	0,92 (0,91–0,94)	-1.590	-7,71
	**Brasil**	**291.375**	**319.561**	**0,91 (0,91–0,92)**	**-28.186**	**-8,82**

Analisando-se em separado as comorbidades selecionadas, foi identificado um padrão oposto, com aumento de óbitos observados para câncer levando a uma RMP de 1,82 (IC95% 1,79–1,85). Um maior número de óbitos observados em relação ao esperado dentro dessa classificação seguiu-se em todas as regiões, com destaque para a região Norte, onde a RMP foi de 2,55 (IC95% 2,37–2,73). Em relação às DCV como comorbidade, também foi constatado excesso de óbitos no Brasil como um todo (RMP = 1,78; IC95% 1,77–1,79) e pelas regiões. A maior RMP também foi vista na região Norte (RMP = 2,46; IC95% 2,41–2,50) (Tabela 2).

**Tabela 2:** Número de óbitos observados e esperados por câncer e por doenças cardiovasculares como comorbidade para a morte e razão de mortalidade padronizada (RMP) no Brasil e regiões entre março e dezembro de 2020.

Comorbidade	Região	Óbitos observados	Óbitos esperados	RMP (IC95%)	Diferença (n)	Variação (%)
**Câncer**	**Norte**	744	292	2,55 (2,37–2,73)	452	154,87
	**Nordeste**	3.776	2.026	1,86 (1,80–1,92)	1.750	86,41
	**Sudeste**	9.928	5.399	1,84 (1,80–1,87)	4.529	83,87
	**Sul**	2.487	1.561	1,59 (1,53–1,66)	926	59,34
	**Centro-Oeste**	1.196	683	1,75 (1,65–1,85)	513	75,09
	**Brasil**	**18.131**	**9.959**	**1,82 (1,79–1,85)**	**8.172**	**82,05**
**Doenças cardiovasculares**	**Norte**	11.651	4.738	2,46 (2,41–2,50)	6.913	145,90
	**Nordeste**	45.298	23.476	1,93 (1,91–1,95)	21.822	92,96
	**Sudeste**	91.923	51.876	1,77 (1,76–1,78)	40.047	77,20
	**Sul**	25.177	17.832	1,41 (1,39–1,43)	7.345	41,19
	**Centro-Oeste**	14.155	7.899	1,79 (1,76–1,82)	6.256	79,20
	**Brasil**	**188.204**	**105.791**	**1,78 (1,77–1,79)**	**82.413**	**77,90**

## DISCUSSÃO

Abrangendo todo o período da pandemia em 2020 e utilizando dados do SIM de todo o Brasil foi possível identificar redução de quase 10% na mortalidade esperada por câncer e por DCV, comparando-se ao ocorrido em 2019. Esse padrão de queda ocorreu nas cinco regiões do país, com uma variação um pouco maior para as DCV entre as regiões.

Uma possível explicação para a redução da mortalidade observada para os dois grupos de doenças aqui estudados é a atuação da covid-19 como causa de morte competitiva, resultando em migração da causa básica da morte. Assim, casos prevalentes de câncer e DCV, que teriam maior risco de óbito por essas doenças, acabaram tendo suas mortes antecipadas em função da covid-19^19^. Estudo realizado na China entre janeiro e junho de 2020, abrangendo três estágios da pandemia (primeira onda, segunda onda e recuperação), demonstrou déficit de mortalidade por todas as causas, porém não apresentou diferença estatisticamente significativa nas mortes por câncer e encontrou excesso nas mortes por DCV^20^. Achados semelhantes, mas que não alcançaram significância estatística, foram relatados no primeiro semestre de 2020 no município de São Paulo para câncer (RMP = 0,9; IC95% 0,67–1,20) e DCV (RMP = 0,9; IC95% 0,68–1,15)^21^.

A competição por leitos hospitalares e atendimento de emergências, bem como o adoecimento de profissionais de saúde, poderia ter levado a um aumento da mortalidade pelas doenças crônicas estudadas. Isso seria esperado em especial para manifestações agudas de DCV, como infarto agudo do miocárdio (IAM), acidente vascular cerebral (AVC) e aneurisma dissecante da aorta. Choudhary et al.^22^ mostraram que o atendimento de emergências cardíacas graves diminuiu durante a pandemia e que, entre os casos atendidos de síndrome coronariana aguda, a proporção de pacientes tratados com terapia conservadora aumentou. Nos EUA, nos meses iniciais da pandemia houve menos internações hospitalares por condições que requerem tratamento de emergência, principalmente IAM, AVC e insuficiência cardíaca^23^.

A redução da mortalidade no Brasil tanto por câncer quanto por DCV em 2020 pode não se manter nos próximos anos. Uma maior dificuldade no acompanhamento e controle de condições crônicas como hipertensão arterial sistêmica e diabetes mellitus deve ter impacto mais tardio no aumento da mortalidade nos próximos anos e medidas para mitigar esse efeito têm sido recomendadas, como o maior uso da telemedicina^24,25^.

Em pacientes oncológicos, o adiamento de cirurgias para remoção de tumores e o atraso no tratamento quimio ou radioterápico podem fazer com que a doença progrida, diminuindo as chances de cura^26^. No Brasil, a pandemia provocou a necessidade de adaptações nas recomendações de detecção precoce, visando priorizar a investigação de casos com sinais e sintomas suspeitos^27^. A redução já percebida dessas ações em 2020^28^ é preocupante por representar impacto futuro na mortalidade por câncer, particularmente em países de baixa e média renda^29^.

Alguns estudos têm se dedicado a estimar, por meio de modelagem, o impacto futuro da pandemia por covid-19 na mortalidade decorrente do retardo no diagnóstico^30^. No Reino Unido, estimou-se que nos próximos cinco anos ocorrerão de 15,3 a 16,6% de mortes adicionais por câncer colorretal e de 7,9 a 9,6% por câncer de mama, de 5,8 a 6,0% por câncer de esôfago e de 4,8 a 5,3% por câncer de pulmão^30^. Na França foi estimado excesso de mortalidade por câncer de mil a seis mil pacientes nos próximos anos^31^. Na Austrália foi previsto impacto importante, em termos de mortes adicionais e custos em saúde, ocasionado por mudanças no estágio da doença no início do tratamento em casos de câncer de mama, pulmão, colorretal (do estágio I para II) e melanoma (do estágio T1 para T2)^32^. Na Índia é esperado excesso de mortalidade relacionada ao câncer do colo do útero^33^.

A pandemia tem sido apontada por alguns como uma oportunidade para reduzir o uso desnecessário e prejudicial dos serviços de saúde, servindo como experimento natural na redução dos danos advindos dessas práticas^34^. É possível que o sobrediagnóstico e sobretratamento, resultantes de rastreamento sem indicação, especialmente para câncer de próstata e de mama, tenham reduzido durante a pandemia, especialmente por ambas serem práticas muito prevalentes no Brasil^35,36^. Embora existam evidências sobre aumento da mortalidade por DCV associada ao sobrediagnóstico e sobretratamento de câncer, e em especial do câncer de mama, é improvável que uma possível redução tenha impactado nos resultados aqui apresentados^37^.

As diferenças observadas entre as regiões brasileiras são reflexo das desigualdades de acesso à saúde, anteriores à pandemia de covid-19^38^. Assim como em outras partes do mundo, com a pandemia, essas desigualdades foram amplificadas^39^, comprometendo as medidas necessárias para controle da disseminação do vírus e evidenciando as dificuldades de acesso aos serviços de saúde. As regiões Norte e Nordeste possuem os menores índices de desenvolvimento humano do país, os maiores índices de desigualdade e o pior acesso aos serviços de saúde^38^. Além disso, suspeita-se que a variante P1 tenha emergido da região Norte^40^, possivelmente contribuindo para o aumento de casos e óbitos no final de 2020 por ser mais transmissível^41^.

A esses fatores, agrega-se ao cenário de pandemia a já alta carga de DCNT nas regiões Norte e Nordeste e descritas desde 2008 como resultantes das desigualdades aqui mencionadas^42^. Ao contrário das demais regiões do Brasil, a tendência da mortalidade por DCV^43^ e por câncer^44^ nessas regiões em anos anteriores à pandemia era de aumento, o que pode ter levado, em especial na região Norte, aos maiores excessos de mortes verificados dessas doenças como comorbidade no período da pandemia.

Em contraste à queda na mortalidade das doenças aqui estudadas como causa básica de óbito no Brasil, observamos aumento expressivo da mortalidade por câncer e DCV como comorbidade, respectivamente 82,1% e 77,9%. Esse aumento variou de 59,3% na Região Sul a 154,9% na Região Norte para câncer e de 41,2% na Região Sul a 145,9% na Região Norte para DCV.

As regras de classificação das causas de óbito no SIM são bem estabelecidas para o câncer^45^ que, em geral, é classificado como causa básica. No caso das DCV a probabilidade de que sejam indicadas como comorbidade no óbito é maior do que no caso do câncer. Com a pandemia, o Brasil passou a seguir o padrão internacional proposto pela OMS para classificação das causas de óbito aplicadas ao SIM^46^, que indica que comorbidades como o câncer não devem ser consideradas como causa básica da morte ainda que tenham contribuído para o agravamento da covid-19, sendo registradas na segunda parte da declaração de óbito.

Os achados deste estudo reforçam uma preocupação importante na pandemia sobre o câncer como comorbidade de doentes com covid-19, a saber, de que o câncer pode afetar o sistema imunológico, fazendo com que pacientes com câncer sejam mais suscetíveis a infecções, seja pelo efeito imunossupressor da quimioterapia e/ou pelo comprometimento da capacidade pulmonar, mais frequente naqueles com câncer de pulmão.

As DCV são reconhecidamente uma das principais comorbidades associadas a um pior prognóstico em pacientes com covid-19^47^ e podem também estar envolvidas como causas antecedentes na cadeia causal do óbito de pacientes com covid-19, aspecto não explorado no presente estudo. Pacientes cardíacos infectados pelo SARS-CoV-2 têm mostrado maior risco de morbimortalidade. O mecanismo fisiopatológico da SARS causada por esse vírus se caracteriza por uma superprodução de citocinas inflamatórias que levam a uma inflamação sistêmica e à disfunção múltipla de órgãos que afeta de forma aguda o sistema cardiovascular^48,49^. A miocardite é uma complicação importante na covid-19^50^. Dos pacientes com covid-19, 7% apresentam injúria miocárdica resultante da infecção, número que sobe para 22% em pacientes graves^51^. Distúrbios nos receptores da enzima conversora de angiotensina (ECA) também apresentam papel importante na patogênese, levando a cardiomiopatia e insuficiência cardíaca^50^.

Hipertensão arterial sistêmica, arritmias, cardiomiopatias e doença arterial coronariana estão entre as principais comorbidades em pacientes com quadro grave de covid-19^50^. Estudo que analisou o papel das DCV como comorbidade e fator de mal prognóstico com dados do SIVEP-Gripe entre pacientes com 20 anos ou mais, hospitalizados com covid-19, confirmado por RT-PCR quantitativo, até agosto de 2020, mostrou que 84% deles tinham uma ou mais comorbidades, incluindo os seguintes tipos de doenças: cardiovasculares, renais, neurológicas, hematológicas, hepáticas, diabetes, doenças respiratórias crônicas, obesidade ou imunossupressão. Esses pacientes apresentaram mortalidade maior quando comparados aos sem comorbidade^52^.

Deve-se destacar como limitação deste estudo o fato de que a base utilizada do SIM para 2020 ainda é preliminar e é possível que as frequências se modifiquem após a consolidação definitiva. Em decorrência da pandemia por covid-19, o Ministério da Saúde antecipou a liberação da base de dados preliminar, prevista pelas normativas para o período entre 30 de junho e 30 de agosto de 2021^53^. Estudos anteriores sobre o impacto da covid-19 na mortalidade no Brasil em 2020 utilizaram a base de dados do Registro Civil^4^ ou o SIVEP-Gripe^52,54^. Essas bases não permitem, contudo, realizar as análises aqui apresentadas por não disponibilizarem a informação detalhada sobre causa básica do óbito ou comorbidades. Por esse motivo, optou-se por utilizar os dados do SIM para 2020, mesmo que preliminares. De fato, observou-se nessa base preliminar que alguns campos, como idade ou data de nascimento, tiveram uma maior quantidade de dados ignorados. O percentual de registros de óbito sem essa informação foi de 0,17% em 2019 passando para 1,98% em 2020. Essa quantidade, contudo, é pequena, não devendo modificar significativamente os achados deste estudo. Da mesma forma, o aumento nos óbitos por causas mal definidas que, após a redistribuição, pode ter contribuído para atenuar a diferença negativa entre os óbitos esperados e observados por câncer e DCV no Brasil e regiões. O incremento dos dados ignorados e de causas mal definidas no período são reflexo da crise sanitária, resultante das dificuldades no acesso aos serviços de saúde e de dificuldades relacionadas ao registro das informações.

Ao considerar a soma dos óbitos por causa básica e comorbidade, a proporção de óbitos onde cada uma delas aparece como comorbidade é inferior à proporção como causa básica para DCV e menor ainda para câncer. No ano de 2020, contudo, houve um claro aumento da citação desses dois grupos de óbito como comorbidade. Para o câncer esperava-se 4,7% e observou-se 9,1%, indicando um aumento de 92%. Entre as DCV, também se constatou um aumento entre o esperado e o observado (24,9% para 39,2%, respectivamente) (dados não apresentados). Essa situação reforça a ideia de que a covid-19 deve ter sido responsável por parte importante de óbitos em pacientes portadores de câncer e de DCV. A pandemia implicou em definições na regra da cadeia de eventos relacionados ao óbito por covid-19, o que pode também ter influenciado o aumento no número de registros do câncer como comorbidade.

## CONCLUSÕES

Os resultados aqui apresentados fornecem uma análise prévia sobre as mudanças no padrão de mortalidade em decorrência da pandemia. O crescimento do número de óbitos citados como comorbidade tanto para câncer como para DCV merece atenção especial e indica necessidade de monitoramento do impacto da covid-19 entre pacientes com essas morbidades. Um destaque deve ser dado à observação de um padrão de aumento das DCV como comorbidades na região Norte durante a pandemia, o que pode ser o resultado de um efeito conjunto da alta carga dessas doenças e menor acesso ao cuidado em saúde. Essas análises são componentes importantes para avaliar e orientar as intervenções e políticas de saúde voltadas para o controle das mortes diretamente ou indiretamente associadas à covid-19.

## Footnotes

a Dados de 2019 disponíveis em ftp://ftp.datasus.gov.br/dissemin/publicos/SIM/CID10/DORES; dados preliminares de 2020 disponíveis em ftp://ftp.datasus.gov.br/dissemin/publicos/SIM/PRELIM/DORES; acessíveis via Internet Explorer ou outro aplicativo para acesso a FTP. Último acesso em 18 fevereiro 2022.

b Instituto Brasileiro de Geografia e Estatística, Sistema IBGE de Recuperação Automática - SIDRA. Projeções da População: projeção da população - edição 2018: tabelas. Rio de Janeiro: IBGE; 2018 [citado 29 abr 2021]. Disponível em: https://sidra.ibge.gov.br/pesquisa/projecao-da-populacao/tabelas
